# Correlates of Light and Moderate-to-Vigorous Objectively Measured Physical Activity in Four-Year-Old Children

**DOI:** 10.1371/journal.pone.0074934

**Published:** 2013-09-05

**Authors:** Esther M. F. van Sluijs, Alison M. McMinn, Hazel M. Inskip, Ulf Ekelund, Keith M. Godfrey, Nicholas C. Harvey, Simon J. Griffin

**Affiliations:** 1 Medical Research Council Epidemiology Unit, Cambridge, United Kingdom; 2 UKCRC Centre for Diet and Activity Research (CEDAR), Cambridge, United Kingdom; 3 Medical Research Council Lifecourse Epidemiology Unit, Southampton, United Kingdom; 4 Department of Sport Medicine, Norwegian School of Sport Sciences, Oslo, Norway; 5 NIHR Southampton Biomedical Research Centre, University of Southampton and University Hospital Southampton NHS Foundation Trust, Southampton, United Kingdom; The University of Texas M. D. Anderson Cancer Center, United States of America

## Abstract

**Background:**

Correlates of physical activity (PA) are hypothesized to be context and behaviour specific, but there is limited evidence of this in young children. The aim of the current study is to investigate associations between personal, social and environmental factors and objectively measured light and moderate-to-vigorous PA (LPA and MVPA, respectively) in four-year-old children.

**Methods:**

Cross-sectional data were used from the Southampton Women’s Survey, a UK population-based longitudinal study. Four-year old children (n = 487, 47.0% male) had valid PA data assessed using accelerometry (Actiheart) and exposure data collected with a validated maternal questionnaire (including data on child personality, family demographics, maternal behaviour, rules and restrictions, and perceived local environment). Linear regression modelling was used to analyse associations with LPA and MVPA separately, interactions with sex were explored.

**Results:**

LPA minutes were greater in children whose mothers reported more PA (vs. inactive: regression coefficient±standard error: 6.70±2.94 minutes), and without other children in the neighbourhood to play with (−6.33±2.44). MVPA minutes were greater in children with older siblings (vs. none: 5.81±2.80) and those whose mothers used active transport for short trips (vs. inactive: 6.24±2.95). Children accumulated more MVPA in spring (vs. winter: 9.50±4.03) and, in boys only, less MVPA with availability of other children in the neighbourhood (−3.98±1.70).

**Discussion:**

Young children’s LPA and MVPA have differing associations with a number of social and environmental variables. Interventions targeting PA promotion in young children outside of formal care settings should consider including intensity specific factors.

## Introduction

Physical activity during childhood is important for health, with active children showing reduced levels of cardiovascular disease risk factors [1], the metabolic syndrome [2] and obesity [3], [4]. Additionally, there is some indication that physical activity in childhood is positively associated with mental health indicators and cognitive performance (leading to better academic achievement) [5], [6]. Promoting physical activity in childhood is therefore a vital step to improving the health and wellbeing of children.

Recently, specific recommendations for physical activity in the under-5s were formulated for the first time internationally, generally encouraging three hours of non-sedentary activity each day [7], [8]. This has been guided by emerging evidence that physical activity has benefits to both physical and psychological health during the preschool years and that it appears to track during early childhood, emphasising the need to establish habits early [9]. However, there is a lack of evidence about the intensity of physical activity most appropriate for health in this age group [8]. In addition, interventions to promote physical activity in young children have generally been sparse and have met with limited success [10]–[12], although effective strategies to promote physical activity during preschool attendance have been identified recently [13], [14].

Relatively little work has been conducted on the putative influences on young children's physical activity. A systematic review reported that 39 factors had been investigated, with the majority only explored in one or two studies [15]. Boys were shown to be more active than girls, and parental physical activity (or parental interaction with child’s physical activity) and time spent outdoors were both positively associated with physical activity. In contrast, age and body mass index (BMI) were consistently found to have no association with young children’s physical activity in this review. An ecological approach to investigating correlates of young children’s physical activity behaviour was called for, which is supported by previous qualitative work with parents of young children [16]–[18]. The socio-ecological model suggests that correlates of behaviour are multi-dimensional and operate at a variety of levels [19]. Variables from the personal, social and environmental domains have all been associated with young children’s physical activity levels [15]. However, few studies in younger children have considered factors from different domains simultaneously. One recent study showed that a variety of factors from the individual, social and environmental domains were independently associated with objectively measured physical activity, although the pattern of associations differed by sex [20]. Exploring the associations between a wide range of variables from different levels and objectively measured activity levels will aid our understanding of which domains are most important for young children’s physical activity levels and help identify avenues for further detailed exploration as well as intervention design.

Data from the population-based sample of the Southampton Women’s Survey (SWS), has previously been used to show that at age four, participants accumulate sufficient activity of any intensity to meet the current physical activity guidelines [4]. However, the majority of this active time was spent in light intensity activity of which the health and developmental benefits are uncertain [4], [8]. In contrast, participants only met the previous guideline (60 minutes of moderate-to-vigorous activity (MVPA) each day of the week) on half of the measurement days (unpublished data). The work presented here set out to further our understanding of how to promote physical activity in this age group, and whether strategies may need to vary depending on the intensity of activity promoted. The aim was therefore to investigate associations between a range of personal, social and environmental factors and objectively measured light physical activity (LPA) and MVPA in four-year-old children. A secondary aim was to explore whether associations differ for boys and girls by means of statistical interaction.

## Methods

### Ethics statement

Ethical approval was obtained from the Southampton and South West Hampshire Local Research Ethics Committee and all participants provided written informed consent for themselves and for their participating four-year old child.

### Study procedures

SWS is a cohort study designed to investigate how women’s anthropometry, lifestyle, and nutrition, before and during pregnancy, affect the development of their offspring [21]. Participants were monitored during their pregnancy and then followed up after the birth of their child. When the children turned four-years old, a sub-sample were invited to attend an additional hospital visit for a secondary study investigating the association between bone health, physical activity and obesity as well as the correlates of physical activity (n = 1,065 invited between March 2006 and June 2009). Data from this four-year visit was utilised for the cross-sectional analysis presented in this paper.

At the four-year visit, study staff measured mothers’ and children’s anthropometry and handed out a previously validated physical activity correlates questionnaire [22]. Children were fitted with an Actiheart monitor (Cambridge Neurotechnology Ltd, Papworth, UK) in order to measure their free-living physical activity. Mothers were asked to return the monitor together with the completed questionnaire by post one week later.

### Outcome variable: physical activity

The Actiheart is a lightweight combined heart rate monitor and accelerometer, previously validated in preschool children [23]. The Actiheart clips onto two ECG electrodes and was positioned in the midline, just below the xiphisternum and attached via a 70−100 mm wire to a smaller clip, horizontally to the left chest wall. Both parts were secured to the skin via standard electrocardiograph electrode pads. Children were asked to wear the monitor continuously for seven days, including during sleep and any water-based activities. To avoid data storage problems during the 7-day assessment, monitors were set to record data every 60 seconds. Methods for interpreting the combined heart rate and movement data are still being developed in young children and consequently only accelerometer data were used. The accelerometer in this device has a linear response to acceleration [24] and was oriented to measure acceleration along the body's longitudinal axis. Accelerometry has been shown to have validity in preschool children [23], [25]. The accelerometer data were analysed using a bespoke program (http://www.mrc-epid.cam.ac.uk/Research/Programmes/Programme_5/InDepth/Programme%205_Disclaimer.html). All recordings between 10pm and 6am were removed as this most likely reflected the hours spent sleeping for the sample (based on the mean counts per minute in these hours). Any periods of ≥100 minutes of zero counts were also excluded [26]. Participants with ≤3 valid days of activity data (defined as ≥10 hours of valid recording) were excluded (n = 48). Included participants provided an average of 5.5 (SD: 0.9) days of valid data with a mean registered time of 15.9 (SD: 0.3) hours, indicating high adherence to the protocol. Time spent in LPA was defined as all minutes with accumulated counts between 20 and 400. Time spent in MVPA was defined using a cut-point of ≥400 counts per minute. The Actiheart cut-points, applying a conversion factor of 5, derived and validated experimentally in children and adolescents [27], [28], roughly equivalent to cut-points of 100 and 2000 from the Actigraph 7164 accelerometer (Actigraph, Pensacola, FL, USA) [29]. Time spent in LPA and MVPA were both correlated with time spent in combined light, moderate and vigorous physical activity (LPA: r = 0.93; MVPA: r = 0.63), but only weakly correlated with each other (r = 0.30).

### Exposure variables

A detailed description of all 31 exposure variables included in the analyses is provided in Table S1.

#### Personal level variables

Five personal level variables were included. Data on children’s sex, height and weight were collected by study staff during the four-year visit. BMI z-score was calculated from the Child Growth Foundation British Growth Charts data [30] using height (in m) measured with Leicester height measures and weight (in kg) assessed using Seca digital scales (to 1 decimal). Data on the remaining three personal variables were collected through the maternal questionnaire.

#### Social level variables

Twenty social level variables were considered, six variables reflecting family demographics, four variables regarding maternal behaviour, and eight variables related to parental rules and restrictions. The last variable represented perceived barriers for physical activity. All were derived from the maternal questionnaire, except for maternal BMI (in kg/m^2^), which was derived from mother’s height and weight measured at the clinic visit. Due to lack of variation in response, the variable indicating whether the father of the four-year-old lived at home was excluded (90.5% yes).

#### Environmental level variables

Five environmental variables were considered, four of which were derived from the maternal questionnaire. The last environmental variable, season, was established by using the first date of measurement.

### Statistical analyses

Comparisons of participant characteristics between those included and excluded from analyses were conducted using chi-squared tests for categorical variables and t-tests for continuous variables.

A two-stage strategy using linear regression analysis was applied to both outcome measures. A combined forwards and backwards stepwise modelling approach was applied. First, unadjusted associations between each potential correlate and LPA and MVPA were assessed. To assess differences in association for boys and girls, interactions with sex were also assessed at this stage. Subgroup analyses were conducted when a significant interaction was observed (p<0.05). Interactions were dropped if no significant association was apparent in either boys or girls. Second, remaining interaction terms and single variables significantly associated at p<0.05 were entered simultaneously into a multiple linear regression model. Non-significant individual variables (and interaction terms) were removed from the multiple model, starting with the variable with the highest p-value until only significant variables and interaction terms remained.

## Results

A total of 730 participants attended the four-year visit between March 2006 and June 2009 when the Actiheart and questionnaire were both distributed. 487 (66.7%) provided both valid physical activity and questionnaire data and formed the sample for this analysis. Participants had a mean±standard deviation (SD) age of 4.1±0.1 years and 47.0% were male. Compared to those excluded from the analysis due to either missing questionnaire or physical activity data (n = 187), those included were more likely to be male (59.7% versus 47.0%, p = 0.003) and to have a higher BMI z-score (mean±SD: 0.47±1.24 vs. 0.11±0.97, p<0.001). No differences were observed for maternal age, maternal BMI or age the mother left full-time education. Descriptive data for all exposure variables are provided in Table 1. On average, children spent 502.6 (SD: 63.8) minutes between 6am and 10pm in LPA and 70.3 (SD: 30.9) minutes in MVPA.

**Table 1:** Distribution of putative correlates of four-year olds’ physical activity (please see Table S1 for a detailed description of the variables).

Variable/Factor	Mean±SD or %
PERSONAL LEVEL	
Sex (%boys)	47.0%
BMI z-score	0.11±0.97
Enjoyment of physical activity	8.9±1.2
Restless	2.4±1.0
Well-behaved	3.9±0.7
SOCIAL LEVEL	
*Family demographic variables*	
Maternal age (yrs)	35.2±3.6
Maternal BMI (kg/m2)	26.6±5.5
Age mother finished education (% >18 years)	33.5%
House ownership (% owning/buying it)	87.4%
Younger siblings (% yes)	45.9%
Older siblings (% yes)	49.4%
*Maternal behaviour*	
Maternal physical activity (score)	2.43±0.98
Maternal screen use (score)	11.8±2.6
Short travel mode (% active)	65.9%
Parental support	15.2±2.7
*Rules and restrictions*	
TV at mealtimes	2.5±1.3
Bedtime	1.6±0.8
Snack at TV	3.0±1.0
PA-related indoor rules	4.0±1.9
Play in garden	3.3±1.3
Restrict computer use	3 (2 to 5)†
Restrict TV watching	4 (3 to 5)†
Restrict playing out	2 (1 to 2)†
*Barriers to physical activity*	
General barriers	8.2±2.8
ENVIRONMENTAL LEVEL	
Environmental barriers	6.9±3.0
Concern about road safety	6.4±2.0
Park availability	4.2±1.1
Other children to play with	3.8±1.2
Season	Winter: 22.8%
	Spring: 25.3%
	Summer: 23.6%
	Autumn: 28.3%

† Median and inter-quartile range presented for skewed distribution.

Table 2 presents the unadjusted and multivariable associations with LPA and MVPA. Five single variables and no interaction terms were taken forward to the multiple model with LPA as the outcome. This subsequently showed that maternal physical activity level was positively associated with four-year olds’ LPA, whereas reporting that there were other children in the neighbourhood to play with was negatively associated. When MVPA was analysed as the outcome, four single terms and three interactions with sex were significantly associated in the unadjusted models. Three single terms and one interaction were retained in the multiple model for MVPA. Results showed that children with older siblings and whose mother used active transport for short trips accumulated more MVPA, and that children were more active in spring compared to winter. In addition, reporting that there were other children in the neighbourhood to play with was negatively associated with boys’ MVPA only. The final models explained 2.4% and 7.9% of the variance in LPA and MVPA, respectively.

**Table 2:** Unadjusted and multivariable associations between potential correlates and four-year-old children’s light physical activity (LPA) and moderate-to-vigorous physical activity (MVPA).

	LPA		MVPA	
Variable	Unadjusted	Multivariable#	Unadjusted	Multivariable#
PERSONAL LEVEL				
Sex (ref: girls)	8.97 (5.79)	-	9.43 (2.77)^**^	NA
BMI z-score	4.79 (2.97)	-	0.12 (1.44)	-
Enjoyment of PA	1.77 (2.53)	-	2.07 (1.22)	-
Restless	2.88 (2.90)	-	1.56 (1.39)	-
Well behaved	−9.09 (4.03)*	NS	−2.92 (1.94)	-
**SOCIAL LEVEL**				
*Family demographic variables*				
Maternal age	−0.38 (0.80)	-	0.39 (0.39)§	-
Maternal BMI	−0.45 (0.53)$	NS	−0.00 (0.25)§	-
Age mother finished education	−9.79 (6.24)	-	−2.34 (3.01)	-
House ownership (ref: renting)	7.15 (8.77)	-	−1.51 (4.24)§	-
Younger siblings (ref: none)	−4.50 (5.80)	-	−1.96 (2.86)	-
Older siblings (ref: none)	4.51 (5.86)	-	6.28 (2.84)*	5.81 (2.80)*
*Maternal behaviour*				
Maternal PA	6.19 (2.94)*	6.70 (2.97)*	0.74 (1.45)	-
Maternal screen use	−0.44 (1.12)	-	−0.44 (0.54)	-
Short travel mode (ref: inactive)	−10.24 (6.14)	-	6.47 (2.95)*	6.24 (2.95)*
Parental support	0.83 (1.07)	-	0.46 (0.52)$	NS
*Rules and restrictions*				
TV at mealtimes	1.82 (2.25)	-	−0.84 (1.09)	-
Bedtime	−0.45 (4.31)	-	−2.92 (2.08)	-
Snack at TV	7.07 (2.97)*	NS	1.60 (1.45)	-
PA-related indoor rules	0.79 (1.55)	-	1.25 (0.75)	-
Play in garden	−3.12 (2.20)	-	1.65 (1.07)	-
Restrict computer use	0.86 (1.22)	-	0.49 (0.59)	-
Restrict TV watching	−1.16 (2.56)	-	−0.44 (1.24)	-
Restrict playing out	6.55 (3.30)	-	0.12 (1.61)	-
*Barriers to physical activity*				
General barriers	1.39 (1.04)	-	−0.26 (0.51)	-
**ENVIRONMENTAL LEVEL**				
Environmental barriers	1.83 (0.99)	-	0.34 (0.48)	-
Concern about road safety	2.95 (1.47)*	NS	1.78 (0.71)*	NS
Park availability	−4.80 (2.67)	-	−1.88 (1.29)$	NS
Other children to play with	−5.96 (2.42)*	−6.33 (2.44)*	−0.55 (1.18)$	G: 1.97 (1.59)
				B: −3.98 (1.70)*
Season (ref: winter)				
Spring	−1.95 (8.38)	-	8.10 (4.02)*	9.50 (4.03)*
Summer	−1.35 (8.51)	-	4.37 (4.09)	6.73 (4.11)
Autumn	−6.15 (8.16)	-	−1.64 (3.92)	−2.24 (3.91)

Numbers in cells are β (SE).

§ A significant interaction (p<0.05) with sex was identified with significant subgroup effects in either boys or girls.

$ A significant interaction (p<0.05) with sex was identified but subgroups effects were non-significant in both boys and girls.

# Where coefficients for boys (B) and girls (G) are reported separately, a statistically significant interaction with sex was retained in the final model.

* : p<0.05; ^**^: p<0.01.

NS: Not significant; NA: not applicable; PA: physical activity; ref: reference category; TV: television.

## Discussion

This study investigated associations between a wide range of personal, social and environmental factors and objectively measured physical activity intensity in four-year old children. Using an exploratory approach guided by the socio-ecological model of behaviour and investigating exposure variables not previously investigated in this age group, it showed that few factors are associated with children’s LPA and MVPA. None of the personal factors or social-level variables related to rules and restrictions were found to be associated, despite considering a range of factors and associations shown previously for pre-school aged and older children [15], [31]. As hypothesized, factors associated with LPA and MVPA were different, with only reported availability of neighbourhood children associated with both. This suggests that varying intervention strategies may be required for the promotion of activity of different intensities and that considering all activity intensities combined may mask important associations.

A novel finding of this study is the positive association between the presence of older siblings in the household and children’s MVPA. Previous work has considered the number of people in the household or number of siblings [20], [32], but the authors are unaware of studies looking at the differential effect of having older and younger siblings. Older siblings may play the role of play-mate, encouraging physically active and rough play, but may also model activity behaviour. The observation that having older siblings is associated with time spent in MVPA, but not in LPA, provides an indication of the intensity of play with older siblings. Interventions should therefore consider the entire family unit, not just the parents and target child, when promoting physical activity [33]. Surprisingly, and in contrast to research in older children [34], maternal reporting of presence of neighbourhood children was negatively associated with LPA and, in boys only, MVPA. The reasons for this unexpected finding are unknown, although it could indicate that interactions with neighbourhood children occur mostly indoors where children are known to be less active [15]. This would fit with a trend towards less independent outdoor activity of children in general [35].

None of the personal level factors were associated with four-year olds’ activity levels, even though some included factors have previously been associated with activity levels in older age groups, such as BMI z-score and enjoyment. In unadjusted analyses, children were engaged in less LPA if their mother reported them to be well behaved. This may reflect the perception that children who sit quietly are better behaved, a potentially desirable attribute of a young child. Although non-significant in the multivariable analyses, it is worth considering the impact of behavioural expectations on children’s activity levels in future work. A mother’s self-reported activity level was positively associated with her child’s time spent in LPA. A positive association between parental and preschooler PA have been consistently shown in previous work [15]. Given the important influence of a mother’s behaviour on her children’s, it is concerning that lower levels of physical activity have previously been observed in mothers of young children compared to women without children [36]. A further understanding of the correlates of mothers’ activity levels is therefore necessary.

This study is the first to consider a mother’s travel choices when travelling with her children as a correlate of children’s activity levels. Interestingly, a third of mothers reported using inactive modes of transport for trips shorter than ½ mile, and their children engaged in less MVPA. It is important to note that the question asked about a mother’s mode of travel, and not her children’s, as these may not be the same. For example, a mother may walk with her preschooler in a buggy. The results therefore do not indicate how *children’s* travel impacts on their activity levels, but it does show that a mother’s choice of travel mode could influence her children’s activity levels either directly (such as children using active transport themselves) or indirectly (through modelling). Further longitudinal research, including whether this may influence uptake of active travel when children grow older, may help elucidate this finding.

In contrast to expectations, none of the variables relating to rules and restrictions regarding physical activity and sedentary behaviour were associated with actual activity levels. However, this is consistent with previous work investigating 15 rules regarding physical activity and sedentary behaviour which only reported one counterintuitive association between restricting rough play inside and higher activity levels in boys [20]. Qualitative evidence highlights the complexity of rules regarding physical activity and sedentary behaviour in this age group, where mothers reported that they were predominantly introduced for children’s safety [17]. In addition, a general lack of heterogeneity in rule-related exposure variables within this age group may partially explain the limited number of associations being observed. Other study designs are therefore needed to further explore the importance of parental rules.

This study considered a large number and broad range of potential exposures, measured across all levels of the socio-ecological model and assessed using a validated questionnaire [22]. Other strengths include the use of an objective measure of physical activity, studying statistical interactions to investigate differences by sex instead of relying on stratified analyses, and the large dataset available. Drop out analyses from the main cohort has previously shown that mothers of children participating at four years were on average slightly older, better educated and smoked less before pregnancy, reducing the generalisability of our findings [37]. For logistical reasons, physical activity monitors were set to 60-second epochs. Given the sporadic nature of young children’s physical activity, shorter epoch lengths have been recommended [25]. The current data does not allow for an investigation of the influence of epoch length on the conclusions drawn. However, the resulting measurement error is unlikely to be differential and will therefore not have led to spurious observations. In addition, the longer epoch length is likely to have resulted in an underestimation of time spent in MVPA [38], and an overestimation of LPA [39], leading to an attenuation of the associations observed. The exposures studied here only explained small proportions of the variance in the outcome. Although this is not uncommon in studies using objectively measured physical activity behaviour as the outcome, it indicates that other factors not measured in our study are simply more important for physical activity in four-year old children. This includes, amongst others, genetic factors, the preschool or care environment, factors related to family members beyond the mother and objectively measured physical environmental features. Finally, we conducted a large number of tests so we cannot rule out the possibility that some of the associations observed are chance findings. However, no adjustment was made for multiple testing to reflect the exploratory nature of the analyses.

## Conclusions

In conclusion, the current study showed that different factors from the social and environmental domains are associated with children’s LPA and MVPA, with few differences between boys and girls. Although the current physical activity guidelines focus on promoting combined LPA and MVPA [7], [8], those designing interventions should consider the intensity of activity to be promoted in order to increase the specificity, and with that the likely effectiveness, of intervention efforts.

## Supporting Information

Table S1 Description and coding of personal, social and environmental variables for putative correlates of physical activity in children from the Southampton Women’s Survey.
(DOCX)
