# Supplementary material for: Correlates of Light and Moderate-to-Vigorous Objectively Measured Physical Activity in Four-Year-Old Children
Source: PLoS One. 2013 Sep 5;8(9):e74934. doi: 10.1371/journal.pone.0074934 (PMC3764204; doi:10.1371/journal.pone.0074934)
Supplement: Table S1 — Description and coding of personal, social and environmental variables for putative correlates of physical activity in children from the Southampton Women’s Survey. (DOCX) [file pone.0074934.s001.docx]

**Table S1:** Description and coding of personal, social and environmental variables for putative correlates of physical activity in children from the Southampton Women’s Survey.

| **Variable/Factor** | **Description/coding** |  |
| --- | --- | --- |
| **PERSONAL LEVEL** | | |
| Sex | % boys | |
| BMI z-score | Body mass index (BMI, kg/m^2^) calculated from measured height and weight, z-scores calculated using standard procedures | |
| Enjoyment of PA | Sum of two questions on whether a mother would describe her child as physically active and whether she feels that her child enjoys physical activity (5-point Likert: strongly disagree–strongly agree). Score: 2-10 (α 0.74) | |
| Restless | Would you describe your child as restless? (5-point Likert: strongly disagree–strongly agree) | |
| Well behaved | Would you describe your child as well behaved? (5-point Likert: strongly disagree–strongly agree) | |
| **SOCIAL LEVEL** | | |
| *Family demographic variables* | | |
| Maternal age | Self-reported date of birth, age calculated using measurement date (in years) | |
| Maternal BMI | Body mass index (BMI, kg/m2) calculated from measured height and weight | |
| Age mother finished education | Age mother finished full-time education (dichotomised as ≤18 years; >18 years) | |
| House ownership | Parent reported house status (own/buying it; rent it) | |
| Younger siblings | Younger children in household (categories: none; yes) | |
| Older siblings | Older children in household (categories: none; yes) | |
| *Maternal behaviour* | | |
| Maternal PA | Index based on physical activity from occupation and during leisure time. Score 1 (inactive) to 4 (active) | |
| Maternal screen use | Sum of 4 questions on time spent watching TV and using computer. Score: 4-24 | |
| Short travel mode | Transport mode when making short trips (<1/2 mile) with child(ren), %active (walk/cycle) | |
| Parental support | Sum of 4 questions on frequency parents watch child do PA, encourage child to do PA, do PA with child and provide transport for child to places where they can be active (5-point scale, never–very often). Score: 4-20 (α 0.75) | |
| *Rules & restrictions* | | |
| TV at mealtimes | Frequency that parents allow child to watch TV at meal times (score 1-5, never–very often) | |
| Bedtime | Frequency that parents allow child to go to bed when they want to (score 1-5, never–very often, latter 3 combined due to low numbers) | |
| Snack at TV | Frequency that parents allow child to eat snacks while watching TV (score 1-5, never–very often) | |
| PA-related indoor rules | Sum of 2 questions on frequency child is allowed to play ball games in the house and run/ride a tricycle in the house (5-point scale, never-very often). Score: 2-10 (α 0.61) | |
| Play in garden | Frequency that parents allow child to play in garden unsupervised by an adult (score 1-5, never–very often) | |
| Restrict computer use | Sum of two questions on how often parents restrict the time child spends using the computer and playing computer games(5-point scale, never-very often). Score: 2-10 (α 0.65) | |
| Restrict TV watching | Frequency that parents restrict the time child watches television (score 1-5, never-very often) | |
| Restrict playing out | Frequency that parents restrict the time child can play outside for (score 1-5, never-very often) | |
| *Barriers to physical activity* | | |
| General barriers | Sum of 4 questions on how often mother feels their child’s PA is limited due to: child not being interested in PA, the weather being too bad, them being too busy, them being scared their child will get hurt(5-point scale, never-very often). Score: 4-20 (α 0.71) | |
| **ENVIRONMENTAL LEVEL** | | |
| Environmental barriers | Sum of 4 questions on how often mother feels their child’s PA is limited due to: fees for PA facilities being too high, it being difficult to get to PA places, there being no parks near home and no other children to play with (5-point scale, never-very often). Score: 4-20 (α 0.65) | |
| Concern about road safety | Sum of agreement with statements that there is heavy traffic in local streets and they are concerned about road safety (5-point Likert: strongly disagree–strongly agree). Score: 2-10 (α 0.64) | |
| Park availability | There are play areas, parks, or gyms close to our home where my child can play (5-point Likert: strongly disagree-strongly agree) | |
| Other children to play with | There are other children near our home with whom my child can go out and play (5-point Likert: strongly disagree-strongly agree) | |
| Season | Season of first day of measurement (Dec-Feb=Winter; Mar-May=Spring; Jun-Aug=Summer; Sept-Nov=Autumn) | |

PA: physical activity; TV: television; α: Cronbach’s alpha
